# Reflections on Peculiar Circumstances Connected with the Recent Occurrence of Exanthematic Typhus in Liverpool

**Published:** 1861-09

**Authors:** Richard J. Dunglison


### Art. II.—*Reflections on Peculiar Circumstances connected with the Recent Occurrence of Exanthematic Typhus in Liverpool.* By Richard J. Dunglison, M.D.

Zymotic diseases are not objects of curious inquiry from their rarity, and it is only, therefore, when the phenomena associated with them present unusual features that we are led again to renew discussions which have long since convinced each side of the incontrovertibility of its own arguments. We could not now, for instance, take up with any show of novelty the theories and views of pathologists as to the intimate cause and nature of typhus fever, or enter into the elaborate argumentative controversies which have occupied so much of the thoughts and the pens of our profession. It is proposed now merely to examine into such points of interest as are attached to the history of a rare instance of the spread of zymotic disease into a locality, as a consequence or the sequence of the visit of individuals affected with other diseases. Cases of this kind offer a wide scope for discussion; so many points arise either at variance with or in consonance with theories long maintained, that at every step we feel we are trespassing on ground already trodden by numerous explorers.

In February last the attention of professional men in the City of Liverpool was specially attracted, by circumstances of unusual interest, to the subject of typhus fever. Without quarantine or precaution of any kind, fever was imported into that great mart of commerce, or rather appeared there as a sequela of the arrival of foreigners covered with filth and vermin, after a sea voyage characterized by overcrowding, exposure, faulty ventilation, and improper food. The details of the history of this visitation, although perhaps already familiar to medical readers, may be again touched upon as essential to a discussion of the points of controversy involved.

The facts are mainly as follows: on the twenty-fifth and twenty-seventh of February, thirty-two Egyptian sailors, forming part of a crew of three hundred, belonging to the Egyptian frigate Scheah Gehald, which had arrived in the Mersey three days before, were admitted to the Southern Hospital. Most of those who were seriously ill were suffering from dysentery or diarrhœa, a few from inflammatory affections of the lungs; the rest were enduring the effects of exposure to cold, which in three instances had occasioned gangrene of some portions of the foot or leg. The voyage from Alexandria had been tedious, and the clothing of the men insufficient. When fresh clothing was procured, each patient was put in a bath and his old linen destroyed. One of the men suffering from dysentery died a few hours after admission; the others were distributed in the different wards.*

Such are the only facts about these men individually which are of any special interest. It is because from them the origination and extension of a hospital endemic is traced that their actual condition upon their arrival is recorded. In about a week after their admission, the house-surgeon complained of sore throat, which in a day or two developed itself into fever; and simultaneously three or four patients in various parts of the building were attacked. Of the Egyptians, twenty-four were discharged cured within the three weeks after admission; and of the remaining eight, three died from dysentery and one from frost-bite.

In connection with the hospital, twenty-four persons took the fever; some of them having it after removal from the hospital. The two house-surgeons had it most severely, and one nurse died of it in the hospital. The pilot of the ship, an attendant in a bathing-place where two hundred

* London Lancet, April, 1861.

apparently healthy Egyptians had bathed, and a patient who had been discharged from the hospital shortly after their admission, died of the disease.

The character of the fever was that of true typhus—at first headache, flushed face, rigors, thirst, deafness, and suffused conjunctivæ. On the sixth or seventh day an exanthematous eruption showed itself on the body, arms, and legs. The pulse rose in some cases to 130 per minute; the tongue gradually coating with a white fur, then, as the disease progressed, becoming brown and dry. In two cases, however, which proved fatal, the tongue kept moist and very little coated, but swollen; and in a third, which recovered, an aphthous eruption appeared on the fauces and back part of the tongue. In most instances the skin was moist after the second or third day, and a tendency to diarrhœa was manifested in nearly all; more or less delirium was also present. In favorable cases the fourteenth day was generally the turning point of the disease, while the fatal cases terminated before that date.

It will thus be observed that none of the Egyptians were affected by the fever, but that they were, *apparently*, the cause of its appearance and extension. Why should they have escaped the fever themselves, and what was the connection between the appearance of dysentery on board ship and of typhus fever on shore? These are points not easily determined; from a single instance like this visit of exanthematic typhus, we can scarcely obtain sufficient data upon which to found many conclusions. Then, too, the profession of the great commercial emporium which formed the locality of the disease seems not to have been awakened to the consciousness of anything unusually interesting occurring among them until the time for collecting minute details had passed, and the outbreak of fever more than attained its acme. In spite of the direct communication with the great metropolis, we find nothing in relation to this febrile invasion in the London medical press until five or six weeks after the admission of the foreign sailors into the Southern Hospital. We are surprised that the outcry sometimes raised in favor of quarantine did not acquire a fresh impetus, when filth and destitution and poverty were thrust upon the attention of the people of Liverpool by these new claimants for their charity, and their careful nursing.

Several questions arise as to the manner in which malignant disease was thus propagated. If we adopt the views of supporters of quarantine regulations, and consider that the fever was imported into Liverpool, we must satisfy ourselves that Egyptians were capable of carrying the seeds of typhus with them, or that such a disease is prevalent in Egypt, from which it had been carried by them in their clothing or their persons. In other words, if the infection was brought from Egypt, is it the form of fever which prevails there? And if they could act as agents of transportation, could these Egyptian sailors live on shipboard, themselves pestiferous and yet not suffering from malignant typhous disease? On the contrary it appears that when the healthy crew returned to Alexandria in another vessel, adynamic fever broke out among them, and was fatal to Egyptians as well as Europeans.

It has been urged by some that typhus fever is never brought to European ports except by vessels coming from places where it was prevalent at the time of their departure. No mention is made in the present instance that it was raging at Alexandria; and, founding our conclusions therefore on the probability of its not being imported from Egypt, we are obliged to fall back upon the view that something on board ship, some pestilential cause, may have operated upon the health of the sailors to produce dysentery, and that these sailors arrived in Liverpool under certain conditions favorable to the propagation of typhus.

We cannot shut our eyes, however, to the fact that Egypt has been frequently visited by pestilence, and its inhabitants sometimes almost decimated by epidemics of the plague. If this disease possessed the characteristics of typhus—and from the description we have by Eusebius of the epidemic of the year 263, the disease alluded to must have been a sort of contagious typhus—we have abundant evidence that the people of Egypt have been subject to a form of typhus fever, and can therefore imagine that the seeds of disease might have been sown at home before the departure of the sailors. But in the absence of any positive testimony on this point, we shall assume, what is much more probable, that they were perfectly free from fever at the time of their embarkation at Alexandria.

It is impossible, of course, when a disease—one of the zymotic class, for example—attacks certain localities in a town, to ascertain exactly in how many of the individuals attacked we can ascribe the invasion to epidemic influences merely, in how many to contact with the infected, or to residence in an atmosphere contaminated by poisonous effluvia. But here we have those not suffering from typhus, but from dysentery, giving rise, apparently, to typhus. Was the dysenteric attack typhus fever in another form, or was it a result of the action of the same poison on shipboard that produced the fever on shore? Is the different sequence of the action of the same poison similar to the result produced at times in hospitals, when patients in the same ward suffer simultaneously from erysipelas, pyæmia, and hospital gangrene? Are the dark races more liable to dysentery than the white?

To answer all these questions satisfactorily it is essential that we should possess a greater amount of materials for information. If we consider the Egyptians as included under the "dark races,"—and we have the authority of works on that country for speaking of certain classes and the inhabitants of certain districts as dark and tawny or bronze,—we may not inaptly quote here the remarks of the distinguished author of the Dictionary of Practical Medicine, Dr. Copland, in favor of the intimate relationship of dysentery and typhus fever.*

"It may be here mentioned that the dark races," he observes, "particularly negroes, are more liable to dysentery than any other disease; that it assumes an extremely low or putrid form in them, when confined in ill-ventilated situations; and that, when a number, even of those in health, are shut up in such places, the cutaneous secretions, which are so abundant and offensive in these races, accumulate in and vitiate the surrounding air, so that if it be not frequently renewed, the systems of those thus circumstanced are thereby infected, and, instead of an infectious typhus, which would be the result in the European constitution, a putrid dysentery, spreading rapidly through all breathing the impure air, is developed. The disease is considered by the native Africans as infectious as small-pox, and is dreaded by them equally with it; these two being the most fatal diseases to which they are liable."

But, if this substitution be true, how did it happen that another set of the same crew, healthy perhaps at starting, as in the trip to Europe, were attacked with adynamic fever on the homeward voyage, and shared the fatality with the Europeans on board? Was not their susceptibility to dysentery as strongly developed and as predisposing to that affection while going from a cooler to a warmer climate, as in their voyage in the opposite direction; had climatic influences anything to do with the change; or were the circumstances altered on board the second ship, so as to prevent the germination of the poison of dysentery? We can only conjecture; but certainly if the dark races are not so frequently liable to low forms of fever as to dysentery, there is no very good reason why, except in the new atmospheric conditions in which they were placed, the same morbid effects should not have supervened.

We find the Egyptian sailors referred to in some of the medical journals as "Turks." The classes of the population of Egypt, however, from which such loathsome objects as the sailors of the Scheah Gehald would be selected, were most probably not of the Turkish element, which, though limited in number, is still the ruling race of the land. But 10,000, according to recent estimates, are Turks and Albanians, out of nearly 2,000,000 souls; and yet the Turkish language is the language of the government, the Turkish portion of the population the dependence of the nation. The victims of the malady are often spoken of as "dark,"

* Vol. i. p. 699.

and we know that the lower classes are darker and coarser than the upper.* If they were Turks in the Egyptian service, they were doubtless liable to the diseases attacking the latter.

In examining into the question whether these Egyptian mariners carried typhus fever with them into Liverpool, we may pertinently ask whether they might not, with equal propriety, be said to have exported the fever when they departed from a place in which, as in the hospital, it was raging? In other words, might we not argue that as they could act, according to the views of some, as conveyers of poison into Liverpool, they could also, even when apparently healthy, with such a wonderful facility for pestiferous communication as has been ascribed to them, produce similar phenomena on shipboard? Dr. Edwards, of Malta, declares that true typhus is never seen there except when imported, thus implying that these sailors must have brought with them the poison, or that the ship was in a favorable condition for the generation of disease.

In the *Medical Times and Gazette* of April 21, 1860, mention is made of outbreaks of fever following the arrival of a vessel, although none of the crew may have been affected. "Missionary Williams," says the writer, "had learned, in the South Sea Islands, to connect the outbreak of pestilential fevers with the arrival of some ship from the coast of Africa, though there might have been no case of fever on board, nor during the voyage, so that he was led to ascribe it to the coming together of different races; and the same was believed to have been the case with H. M. S. *Growler*, with free laborers from the coast of Africa to the West Indies, in 1847. And thus, to point to the former history of the Egyptian frigate as an important part of the matter for investigation; for, if it be that there never has been a fever of the kind on board before, then, perhaps, Missionary Williams came nearest the truth after all in saying that 'contagion' or 'infection' is conveyed in, and given off from, the filthy skin of living persons."

Was the sickness which resulted in admission into the Southern Hospital well-marked dysentery, or was it typhus fever in another form, its exanthematic type hidden from the observation and diagnostic powers of medical men by the dark color of the skin? It is sometimes a plausible method of getting over difficulties of theories by leaving the main points of attack, and, by a flank movement, taking a new position which may become at once untenable again. It would be little to the credit of Liverpool medical diagnosis, had an adynamic disease in any form escaped notice, merely because it occurred in a darker race than that which they had been accustomed to see daily in their private and hospital practice.

* English Cyclopædia, Geography, vol. ii. Lond., 1854, p. 885.

Besides, the cases of dysentery admitted immediately after the arrival of the Egyptian vessel remained long enough in the hospital—in fact, some days after the outbreak of typhus—to be studied in connection with the fever; and no description that we have seen of both sets of cases exhibits similarity of phenomena sufficient to warrant the assumption that they were forms of the same disease,—special subdivisions under the one general head, Typhus. Assuredly, the adynamia or the dysentery must have predominated, and it is scarcely probable that by a curious coincidence the latter should have been so much in excess in all the darker subjects, and the former in all the fresher cases occurring in the hospital among the whites.

One of the medical journals,* in its comments on this remarkable attack of typhus fever, and the important evidence it may afford as to its cause, remarks, that several points should be accurately determined before any positive conclusions are arrived at. Is it true, as asserted, that no single person among the Turkish (? Egyptian) crew had typhus at the time when the disease was communicated to persons in Liverpool? Had the Scheah Gehald any cases of typhus fever during the voyage, or had any of the crew any communication with persons ill of that disease? Can it be clearly made out that *all* the cases occurring at Liverpool among the inhabitants were really cases of exanthematic typhus? Did the disease seem to be caused by emanations from the *clothes* or from the *persons* of the Turkish sailors, who were dirty and sick, but who, it was stated, had not typhus? Thousands of Turks are said, by the same writer, to have died of the disease during the allied war in the Crimea; but if these were not Turks but Egyptians, the pertinence of the remark is not so striking. This confounding of different races does not materially add to the clearness of the discussion, or give importance to the arguments of those who practice it. We know little of the diseases and hygienic condition of the lower classes of the Egyptians, but we should not on that account appeal to the history of the small Turkish element in the upper classes of society there for all our knowledge of Egyptian pathology and practice of medicine.

We have already mentioned that those of the crew of the Scheah Gehald who returned home in the frigate *Voyageur de la Mer* had suffered from a form of adynamic fever. Dr. Edwards, of Malta, does not consider such cases as came under his observation, at that intermediate station, illustrations of the peculiar typhus fever which he had been accustomed to see in Europeans. And yet the symptoms he describes, if not associated with typhus so called, are characteristic of a low type of disease. The

* Medical Times and Gazette, April 13, 1861, p. 390.

pulse was quick, over 100 in every case; the tongue white and swollen; they looked stupid, miserable, and wo-begone; they had watery eyes and great debility and headache. The Englishmen who suffered on board the same vessel had similar symptoms, so far as pulse, tongue, and conjunctivæ were implicated, but manifestations of extreme cerebral disorder were superadded, such as deafness, low-muttering delirium, tremor of the lower jaw in speaking, with copious mulberry rash, and afterward sudamina.

We have here another phase of the question of nationality as influencing the spread of zymotic diseases. It seems to us that the cases among the Egyptians might have been examples of typhus in an early stage of its development, beyond which, for some unknown cause, the disease could not progress. And yet it is strange that all the Orientals who were attacked with the fever should be unable to advance beyond the limits of a single stage, when exposed to similar active causes of disease with the Europeans, while the latter passed with apparently the greatest facility into the more developed forms of the fever.

Whatever may have been the cause of this difference of susceptibility, whether nationality, difference of exposure, or other circumstances, or whatever the name by which we may distinguish the affection of which they were the victims, the mortality among the Egyptians during the passage to Malta was great. Why, if the fever was less adynamic with them, should the mortality have been serious? It is mentioned that some of those who were sick came on board in that condition at Liverpool, but it is not stated with what special complaint they were afflicted. If with dysentery, the previous question recurs as to the mutual influence of typhus fever and dysentery. If with typhus fever, the medical authorities at Liverpool and on board the steamer are reprehensible for their laxity of medical discipline and precaution. If with any other disease, was their attack of adynamic fever associated with, or independent of their other sufferings? In every view in which we study the history of this whole subject, new points of interest arise to occupy, puzzle, and confuse us.

Could quarantine regulations have prevented the spread of zymotic disease in this instance?

If it originated from the ship or crew, the purifying effects of personal cleanliness and ventilation might have been most effective, and in all such cases it would be well to have recourse to such sanitary precautions. Where persons have been crowded together for a time, and adynamic disease of any kind has appeared among them, the animal exhalations accumulate in such quantity in the apartments and about the person, while there has been an absence of proper ventilation and cleanliness, as in the case of the Black Assizes mentioned hereafter, as to act frequently like true ferments, and endanger the production of adynamic zymotic disease of some kind.

In the case in question, however, there was no marked indication in the first instance for the establishment of any rigorous quarantine, or of isolation of any kind. Still, all the circumstances should be borne in mind, and even should doubt exist of the general efficacy of such restraint upon commerce, when circumstances of an analogous kind present themselves, there ought to be none as to the propriety of adopting a thorough system of ventilation and cleansing of both the ship and the crew.

And now, after studying the history of this invasion of fever, the absence of exanthematous typhus in those who were apparently the causes of its appearance, the importation of low forms of fever, the prevalence of epidemic pestilential diseases in their own country, from which these Egyptians are presumed by some to have brought it, the diseases of the darker races, the connection between typhus and dysentery, the adynamic fever of the return voyage, the ethnology of the Turkish element in the Egyptian population, the association of outbreaks of fever with the arrival of certain vessels, the points necessary to determine at the outset of every investigation into the pathology of pestilential diseases, and the effects of quarantine regulations, we may finally endeavor to ascertain the true causes of the appearance of typhus fever at the time and at the place in which it actually occurred.

Very little stress is laid, by those who have referred to the fever in the medical journals, upon the fact that typhus was endemic in the immediate vicinity of the hospital at the very time when a new disturbing element was brought to the spot in the persons or the clothes of the filthy Oriental sailors. It is easy to understand that under other circumstances less favorable, these same filthy foreigners might have entered Liverpool, might have been thoroughly purged of their uncleanness, and have resided there or left there without any fatal consequences ensuing to those around them. It is not filth and faulty ventilation and crowding that constitute the sole predisposing causes of typhus, for all these conditions have existed over and over again without producing it. It is when favorable circumstances exist for its development in addition to all these, such as are found in atmospheric vicissitudes, personal exposure, etc., that the appearance of low forms of fever is so apt to follow.

We do not know when, therefore, to expect typhus to occur, merely because crowds are collected together in ill-ventilated places, but we have a ready explanation always at hand when these favorable conditions exist. So it may have been in the present instance; with a locality predisposed to a dangerous malady; an incursion of filth and squalor; no care employed to remedy the new evils which were thus superimposed upon the old; and no separation of the sick already in the hospital from the newcomers; it is not necessary to appeal to any connection between dysentery and typhus, or the substitution of the one for the other in different races.

Had several hundred of the lowest classes of the Liverpool population deserted for a time the discomforts of their own homes, and the poverty of their own resources, and after a life of toil on shipboard, surrounded by all the unfavorable circumstances of effluvia and atmospheric poisoning under which the poor Egyptians made their long passage over the ocean, these Englishmen, though not dark, might not the less have suffered from dysenteric attacks, might have arrived in Alexandria and been sent to a hospital, and have been the agents by which ataxic fever should originate and be diffused.

We are told by one writer on the subject, Dr. Cameron, Physician to the Liverpool Southern Hospital, that typhus was endemic in Liverpool, and that one of its favorite haunts was the locality in which the hospital was situated,—a fact of so much importance that we are surprised that it has excited so little notice in the discussion.

The details of the attack of exanthematic typhus which have now been fully dwelt upon, recall the history of a celebrated event in the annals of English jurisprudence. An analysis of the medical facts connected with the "Black Assize," as the fatal assize held in 1577, in Oxford town-hall, is called, presents some similarity of phenomena with that which we have been discussing. Persons living in an infected locality, not suffering from disease themselves, were, in each case, transferred to other spots, in which apparently healthy persons soon became the subjects of infectious disease. The facts of this point in Oxonian history may not be familiar to all. The best account of it is given in Anthony à Wood's "History and Antiquities of the University," published in 1796, vol. ii. p. 188, in which, speaking of the trial of one Jencks for sedition, it is remarked: "Judgment being passed, and the prisoner taken away, there arose such an infectious damp or breath among the people, that many there present, to the apprehensions of most men, were then smothered, and others so deeply affected that they lived not many hours after. The persons that then died, and were infected by the said damp, when sentence was passed, were Sir Robert Bell, baron of the Exchequer; Sir Nicholas Barham, sergeant-at-law; Sir Robert d'Oyley, the high sheriff; Hart, his under-sheriff; Sir William Babyngton, Robert d'Oyley, Wenman, Danvers, Fetiplace, and Harcourt, justices of the peace; Kerle, Greenwood, Nash, and Forster, gentlemen; besides most of the jury, with many others that died within a day or two after. Above 600 sickened in one night, as a physician of Oxford* attested; and the day after, the infectious air being carried into the next villages, there sickened 100 more. The fifteenth,

* Geog. Edrycus in "Hypomnematibus Suis in Aliquot libros Pauli Æginetæ," edit. London, 1588, lib. 2.

sixteenth, and seventeenth days of July, sickened also above 300 persons, and within twelve days' space died 100 scholars, besides many citizens. The number of persons that died in five weeks' space—namely, from the sixth of July to the twelfth of August (for no longer did this violent infection continue)—were 300 in Oxford, and two hundred and odd in other places; so that the whole number that died in that time were 510 persons, of whom many bled till they expired. Some, the writer states, 'left their beds, occasioned by the rage of their disease and pain, and would beat their keepers or nurses, and drive them from their presence. Others ran about the streets and lanes in a state of frenzy, and some even leaped headlong into deep waters. * * * The parties that were taken away by this disease were troubled with a most vehement pain of the head and stomach, vexed with the frenzy, deprived of their understanding, memory, sight, hearing, etc. The disease also increasing, they could neither eat nor sleep. * * * At the time of their death, they would be very strong and vigorous; but if they escaped it, then they were to the contrary. It spared no complexion or constitution, and the choleric it chiefly molested. That which is most to be admired is, that no women were taken away by it, or poor people, or such that administered physic, or any that came to visit. But as the physicians were ignorant of the causes, so also of the cures of this disease.'" The same authority mentions a similar event at Cambridge, at the assizes held in the castle there in the time of Lent, 13 Henry VIII., A.D. 1521, where the justices, all the gentlemen, bailiffs, and most who resorted thither, took such an infection that many of them died, and all almost that were present sickened, and narrowly escaped with their lives.*

---

* English Cyclopædia, conducted by Charles Knight, Arts and Sciences, vol. ii., London, 1859, p. 178.

